# Baicalin Inhibits FIPV Infection In Vitro by Modulating the PI3K-AKT Pathway and Apoptosis Pathway

**DOI:** 10.3390/ijms25189930

**Published:** 2024-09-14

**Authors:** Zhongda Cao, Nannan Ma, Maoyang Shan, Shiyan Wang, Jige Du, Jia Cheng, Panpan Sun, Na Sun, Lin Jin, Kuohai Fan, Wei Yin, Hongquan Li, Chunsheng Yin, Yaogui Sun

**Affiliations:** 1 Shanxi Key Laboratory for Modernization of TCVM, College of Veterinary Medicine, Shanxi Agricultural University, Jinzhong 030801, China; caozhongda_sc@163.com (Z.C.);; 2 China Institute of Veterinary Drug Control, Beijing 100081, China; 3 Laboratory Animal Center, Shanxi Agricultural University, Jinzhong 030801, China

**Keywords:** baicalin, feline infectious peritonitis, network pharmacology, molecular docking, proteomics

## Abstract

Feline infectious peritonitis (FIP), a serious infectious disease in cats, has become a challenging problem for pet owners and the industry due to the lack of effective vaccinations and medications for prevention and treatment. Currently, most natural compounds have been proven to have good antiviral activity. Hence, it is essential to develop efficacious novel natural compounds that inhibit FIPV infection. Our study aimed to screen compounds with in vitro anti-FIPV effects from nine natural compounds that have been proven to have antiviral activity and preliminarily investigate their mechanisms of action. In this study, the CCK-8 method was used to determine the maximum noncytotoxic concentration (MNTC), 50% cytotoxic concentration (CC_50_), and 50% effective concentration (EC_50_) of natural compounds on CRFK cells and the maximum inhibition ratio (MIR) of the compounds inhibit FIPV. The effect of natural compounds on FIPV-induced apoptosis was detected via Annexin V-FITC/PI assay. Network pharmacology (NP), molecular docking (MD), and 4D label-free quantitative (4D-LFQ) proteomic techniques were used in the joint analysis the mechanism of action of the screened natural compounds against FIPV infection. Finally, Western blotting was used to validate the analysis results. Among the nine natural compounds, baicalin had good antiviral effects, with an MIR > 50% and an SI > 3. Baicalin inhibited FIPV-induced apoptosis. NP and MD analyses showed that AKT1 was the best target of baicalin for inhibiting FIPV infection. 4D-LFQ proteomics analysis showed that baicalin might inhibit FIPV infection by modulating the PI3K-AKT pathway and the apoptosis pathway. The WB results showed that baicalin promoted the expression of EGFR, PI3K, and Bcl-2 and inhibited the expression of cleaved caspase 9 and Bax. This study found that baicalin regulated the PI3K-AKT pathway and the apoptosis pathway in vitro and inhibited FIPV-induced apoptosis, thus exerting anti-FIPV effects.

## 1. Introduction

FIP, a serious infectious disease in cats, can cause symptoms such as fervescence, anorexia, weight loss, dyspnea, and diarrhea. This is a systemic and fatal infectious disease [1]. FIP is caused by infection with feline infectious peritonitis virus (FIPV) [2], which is commonly observed in kittens aged 6–24 months and older cats aged over 11 years, and usually leads to the failure of various tissues and organs of the body due to coinfection with multiple diseases, eventually leading to death [3]. This is a serious hazard to the pet industry. Currently, there is no effective vaccine for the prevention of FIPV [4], and drug treatment mainly includes symptomatic treatment and treatment with the nucleoside analog GS-441524, but the treatment effect is unsatisfactory [5]. Therefore, finding novel medications that may effectively inhibit FIPV infection is imperative.

Some natural compounds extracted from herbal medicines have become the first choice for new drug development and screening by many researchers due to their widespread presence in nature and low drug resistance. There are also a number of natural compounds that have been shown to have significant effects on inhibiting viral infections. For instance, baicalin and chlorogenic acid can inhibit the infection of chicken embryo infectious bronchitis virus (IBV) [6,7]; matrine can inhibit the infection of PRRSV [8]; caffeic acid can significantly inhibit the invasion of hepatitis C virus (HCV) into cells [9]; glycyrrhizic acid exhibits dose-dependent inhibition of Epstein-Barr virus (EBV) [10]; and puerarin, tanshinone IIA, and dihydrotanshinone I all have inhibitory effects on SARS-CoV-2 infection [11,12,13].

Therefore, to identify the natural compounds that inhibit FIPV infection, we used CRFK cells to screen the above nine natural compounds with antiviral activity in vitro. Then, we studied the mode of action of the compounds that inhibit FIPV infection and their effects on FIPV-induced apoptosis and combined our findings with the results of network pharmacology (NP) [14], molecular docking (MD) [15], and 4D label-free quantification (4D-LFQ) proteomics [16] technology analyses, which are popular in current mechanistic studies, to explore the mechanism of natural compounds against FIPV infection and lay the foundation for the research and development of new drugs and the in-depth exploration of the mechanism of action.

## 2. Results

### 2.1. In Vitro Screening of Nine Natural Compounds Revealed That Baicalin Can Inhibit FIPV Infection

**Figure 1:**
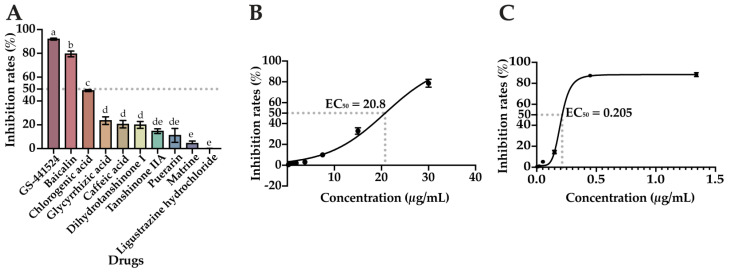
These compounds inhibited FIPV infection: (**A**) inhibition rate of the compounds against FIPV infection; (**B**) concentration-inhibition curve of FIPV treated with baicalin; (**C**) concentration-inhibition curve of FIPV treated with GS-441524. The data are represented as the mean ± SEM (*n* = 3). Different lowercase letters (a–e) indicate significant differences between groups (*p* < 0.05).

Based on the CPE of each compound on the cells and the data obtained from the CCK-8 assay, the MNTC and CC_50_ of each compound on CRFK cells were determined using the CR calculation formula, and the results are shown in Table 1. According to the results of the CPE and the data measured via the CCK-8 method, stoichiometric dependence was observed for all nine compounds tested and for the positive drug (GS-441524), and as the concentration of the compound increased, the degree of CPE of the cells also increased: the cells became round, clustered, and detached. The results of the coaddition of the compounds to be tested and FIPV to a 96-well cell culture plate for incubation are shown in Figure 1A. The positive drug GS-441524 inhibited FIPV significantly more than the other compounds tested (*p* < 0.05), and among the nine compounds tested, dihydrotanshinone I, tanshinone IIA, puerarin, matrine, and ligustrazine hydrochloride inhibited FIPV infection by less than 20%; the inhibition of FIPV infection by chlorogenic acid, glycyrrhizic acid, and caffeic acid ranged from 20 to 50%; and only baicalin inhibited FIPV by 79.5% (more than 50%); its inhibition rate was significantly greater than that of the other compounds tested (*p* < 0.05). Afterwards, a gradient dilution of baicalin and GS-441524 were used to inhibit FIPV infection, respectively; and the EC_50_ of baicalin and GS-441524 were calculated to be 20.8 μg/mL and 0.205 μg/mL, respectively, by GraphPad Prism 10 (Figure 1B,C). The SI of baicalin was calculated to be 5.5 by the formula, which met the screening conditions (MIR > 50%, SI > 3). Therefore, baicalin was used for subsequent tests.

### 2.2. Impact of Baicalin on FIPV Replication, Adsorption, and Its Direct Inactivation of FIPV

**Figure 2:**
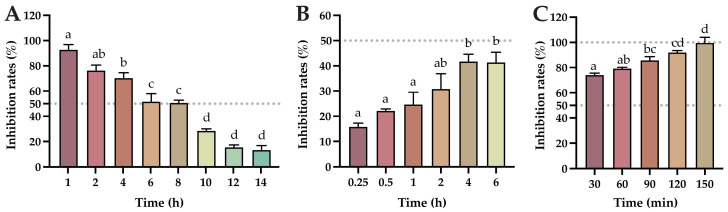
The impact of baicalin on FIPV replication, adsorption, and its direct inactivation of FIPV: (**A**) the effect of baicalin on FIPV replication; (**B**) the effect of baicalin on FIPV adsorption; (**C**) results of direct inactivation of FIPV by baicalin. The data are represented as the mean ± SEM (*n* = 3). Different lowercase letters (a–d) indicate significant differences between groups (*p* < 0.05).

From the results of the determination of the viral replication blocking effect of baicalin (Figure 2A), the inhibition rate of baicalin on FIPV gradually decreased with the increase in the time of FIPV infection of CRFK cells, and baicalin had more than 50% inhibition of FIPV infection of CRFK cells within 8 h; the inhibition rate was significantly reduced after 10 h of infection (*p* < 0.05). These results indicate that baicalin had a good inhibitory effect on viral replication within 8 h of FIPV virus infection, with an inhibition rate of more than 50%. From the results of the effect of baicalin on FIPV adsorption (Figure 2B), the inhibition rate of baicalin on FIPV infection increased with the increase in the co-culture time of baicalin and cells, but none of them exceeded 50%. These results indicate that baicalin was not effective in inhibiting FIPV adsorption. From the results of the direct inactivation effect of the compound on FIPV (Figure 2C), when baicalin was added to CRFK cells after coculturing with FIPV for a period, the inhibition rate of baicalin on FIPV increased with the increase in the time of co-culturing, and all of the inhibition rates were higher than 50%. At 150 min of co-culture, the inhibition rate of baicalin on FIPV infection tended to be 100%. These results indicated that baicalin had a direct inactivation effect on FIPV and showed a time-dependent effect.

### 2.3. Baicalin Inhibits FIPV-Induced Apoptosis Network Pharmacology and Molecular Docking Analyses Identify AKT1 as the Best Target of Action for Baicalin against FIPV Infection

**Figure 3:**
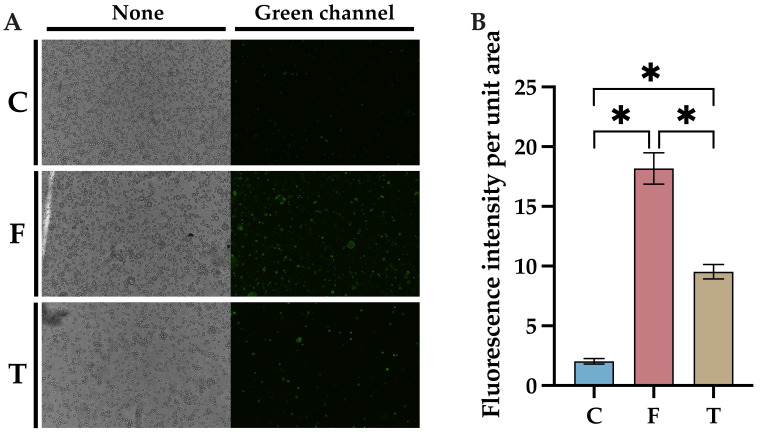
Effects of baicalin on FIPV-induced apoptosis: (**A**) results of fluorescence microscopy observation with a magnification of 100×; (**B**) results of comparison of fluorescence intensity per unit area of cells. C: cell control group; F: virus control group; T: baicalin-treated group. The data are represented as the mean ± SEM (*n* = 3). * *p* < 0.05.

A baicalin-resistant FIPV-infected CRFK cell model was generated, and apoptosis was detected using the Annexin V-FITC/PI assay 24 h after FIPV infection. More green fluorescence, representing early apoptosis, was detected in the virus control group than in the control group, while significantly less green fluorescence was observed in the baicalin-treated group than in the virus control group (Figure 3A). However, red fluorescence representing late apoptosis was not observed, probably due to the short duration of infection. The fluorescence intensity per unit area of cells was calculated using ImageJ, and the results showed that the fluorescence intensity per unit area of cells in the baicalin-treated group was significantly lower than that in the control group (Figure 3B, *p* < 0.05), which indicated that baicalin can inhibit FIPV-induced apoptosis.

### 2.4. NP and MD Analyses Showed That AKT1 Is the Best Target of Baicalin for Inhibiting FIPV Infection

**Figure 4:**
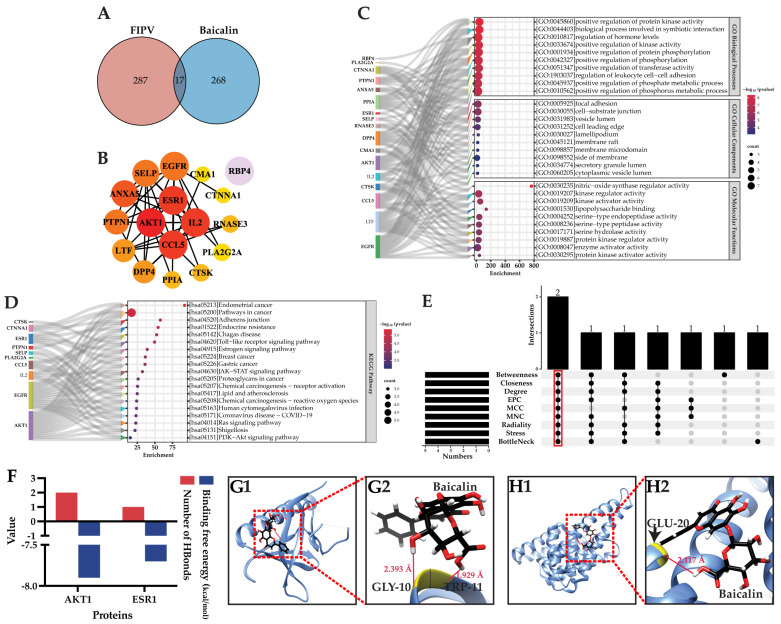
(**A**) Venn diagram of baicalin target-FIPV targets; (**B**) PPI network of potential targets; (**C**) Sankey bubble plot of GO functional enrichment results; (**D**) Sankey bubble plot of KEGG pathway enrichment results; (**E**) results of topological analysis; (**F**) results of molecular docking binding free energy and number of hydrogen bonds; (**G1**,**H1**) overall maps of baicalin docked with AKT1 and ESR1, respectively; (**G2**,**H2**) local maps of baicalin docked with AKT1 and ESR1, respectively.

A total of 17 potential targets were obtained via the intersection screening of 285 baicalin-interacting targets obtained from PharmMapper and 304 FIPV-pathogenic-interacting targets obtained from GeneCards (Figure 4A, Table 2). These 17 targets are potential targets by which baicalin inhibits FIPV infection. These potential targets were uploaded to STRING to construct a protein-protein interaction (PPI) network with 17 nodes, 43 edges, 5.06 average node connections, 0.656 average local clustering coefficient, 16 expected edges, and a PPI-enriched *p*-value of less than 3.63 × 10^−8^. Node information was imported into Cytoscape; the colors of nodes were sorted according to the degree; and the PPI network of potential targets was finally obtained. These results showed that RBP4 was not connected with the other targets (Figure 4B). Metascape was used to analyze the GO function and KEGG pathway enrichment of potential targets, and the results showed that a total of 230 GO functional enrichment results were obtained, of which 182 were for biological processes, mainly in the positive regulation of protein kinase activity, the regulation of leukocyte cell-cell adhesion, and the positive regulation of transferase activity, etc.; 18 were for cellular components, mainly concentrated in cellular components such as focal adhesion, vesicular lumens, cell leading edges, lamellipodium, and membrane microdomain, etc.; and 30 were for molecular functions, mainly enriched in kinase regulator activity, kinase activator activity, lipopolysaccharide binding, and protein kinase regulator activity, etc. The top 10 results of the GO functional enrichment in each category are shown in Figure 4C. KEGG pathway enrichment analysis revealed a total of 19 pathways (Figure 4D), which were enriched mainly in the Toll-like receptor signaling pathway, the JAK-STAT signaling pathway, the Ras signaling pathway, and the PI3K-AKT signaling pathway. Nine topological analyses of potential targets were performed using CytoHubba 0.1, and the results showed that two targets were co-ranked in the top five of each analysis method, and these were the key targets, which were AKT1 and ESR1 (Figure 4E).

The MD of AKT1 and ESR1 with baicalin showed that the binding free energy of baicalin-AKT1 was −7.9 kcal/mol, and it formed two hydrogen bonds. The binding free energy of baicalin-ESR1 was −7.7 kcal/mol, and the number of hydrogen bonds formed was one. Compared with baicalin-ESR1, baicalin-AKT1 has a lower binding free energy and can form a stable complex (Figure 4F). According to the molecular docking model, baicalin formed hydrogen bonds with GLY-10 (glycine) and TRP-11 (tryptophan) of AKT1 (PDB ID: 1UNR), with bond lengths of 2.393 Å and 1.929 Å, respectively. Baicalin formed a hydrogen bond with GLU-20 (glutamic acid) of ESR1 (PDB ID: 7BAA), with a bond length of 2.117 Å (Figure 4G–H). According to the docking results, the best docking target of baicalin was AKT1. In conclusion, AKT1 is the best target of baicalin for inhibiting FIPV infection.

### 2.5. Determination of the Cellular Viral Load at Different Times of Baicalin Action

**Figure 5:**
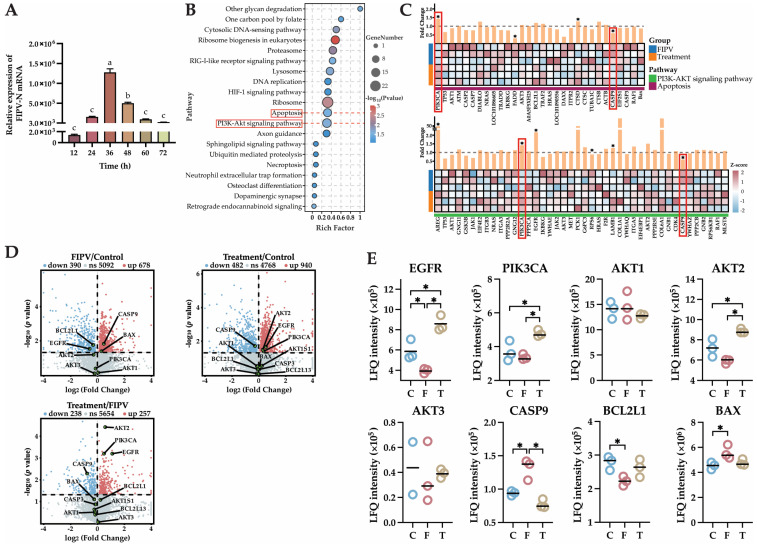
(**A**) results of viral load in cells treated with baicalin for different durations. (**B**) KEGG pathway enrichment bubble map. (**C**) Heatmap and fold change map of the PI3K-AKT signaling pathway and apoptosis pathway. (**D**) Volcano plots of DEPs in the three comparative groups of FIPV/control, treatment/control, and treatment/FIPV, in which the key proteins EGFR, PIK3CA, AKT1, AKT2, AKT3, CASP9, BLC2L, and BAX in the PI3K-AKT signaling pathway and apoptosis pathway were locally annotated with green dots. (**E**) 4D-LFQ proteomics quantitative analysis results of the key proteins in the PI3K-AKT signaling pathway and apoptosis pathway: EGFR, PIK3CA, AKT1, AKT2, AKT3, CASP9, BLC2L1, and BAX. The red boxes are the focus of this research. The data are represented as the mean ± SEM (*n* = 2–3). Different lowercase letters (a–c) and “*” indicate significant differences between groups, *p* < 0.05.

qPCR was used to detect the viral load of FIPV-infected CRFK cells at 12, 24, 36, 48, 60, and 72 h after baicalin treatment. The FIPV-*N* gene copy number peaked at 36 h after baicalin treatment (*p* < 0.05) and significantly decreased at 48 h compared with that at 36 h (*p* < 0.05); therefore, the collection of cell model samples was performed after 48 h of baicalin treatment (Figure 5A).

### 2.6. The 4D-LFQ Proteomics Analyses Suggested That Baicalin May Inhibit FIPV Infection by Regulating the PI3K-AKT Signaling Pathway and the Apoptosis Pathway

**Figure A1:**
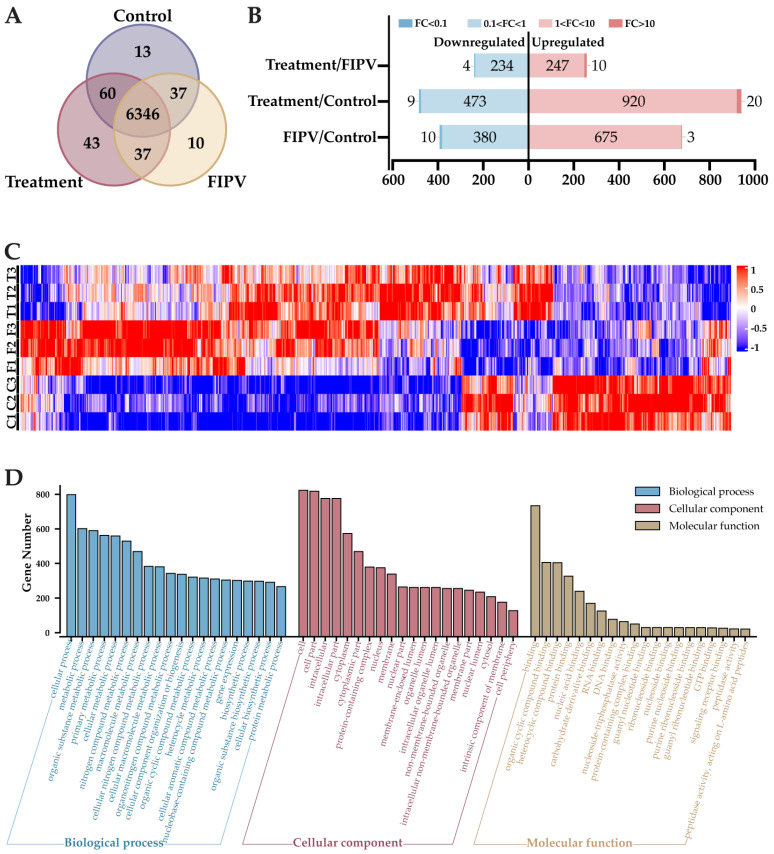
Results of proteomic analysis: (**A**) Venn diagram of proteins detected in control, treatment, and FIPV groups; (**B**) DEPs among the treatment/FIPV, treatment/control and FIPV/control groups; (**C**) DEP clustering results of groups C, F, and T. Control or C is the cell control group; treatment or T is the baicalin-treated group; and FIPV or F is the viral control group, the same as below. (**D**) GO functional enrichment results.

A total of 6546 proteins were detected via 4D-LFQ proteomics, among which 6456, 6430, and 6486 proteins were detected in the control group, FIPV group, and treatment group, respectively, for a total of 6346 proteins among the three groups (Figure A1A). The number of differentially expressed proteins (DEPs) and their up- and downregulation among the comparison groups are shown in Figure A1B. There were 257 upregulated proteins and 238 downregulated proteins in the treatment/FIPV group; 940 upregulated proteins and 452 downregulated proteins in the treatment/control group; and 678 upregulated proteins and 390 downregulated proteins in the FIPV/control group. Cluster analysis after Z score normalization of the above-detected DEPs showed that there was a difference between the groups of samples in this proteomics assay (Figure A1C).

The results of the GO functional enrichment analysis of the DEPs are shown in Figure A1D. Biological process (BP) terms were enriched mainly in cellular process, metabolic process, cellular biosynthetic process, and protein metabolic process. Cellular component (CC) terms were enriched mainly in the cell part, cytoplasm, intracellular organelle lumen, and nuclear lumen. Molecular function (MF) terms were enriched mainly in organic cyclic compound binding, carbohydrate derivative binding, RNA binding, and DNA binding.

KEGG pathway enrichment results showed that the DEPs were mainly enriched in apoptosis, DNA replication, the PI3K-AKT signaling pathway, the HIF-1 signaling pathway, the RIG-I-like receptor signaling pathway, and the cytosolic DNA-sensing pathway. (Figure 5B). Combined with the results obtained in 2.3 and 2.4 of this study (baicalin can inhibit FIPV-induced apoptosis, and AKT1 is the best target of baicalin for inhibiting FIPV infection), the PI3K-AKT signaling pathway and the apoptosis pathway were selected for analysis (red box content in Figure 5B). Both pathways were in the top 20 according to the *p*-value ranking, and the number and proportion of DEPs were similar between them. Heatmaps drawn from the DEPs in the treatment/FIPV comparison group showed that PIK3CA and CASP9 were present in both pathways (red boxed sections), with PIK3CA showing significant upregulation between the two pathways (*p* < 0.05) and CASP9 showing significant downregulation between the two pathways (*p* < 0.05, Figure 5C). Volcano plots from two-by-two comparisons of the control, treatment, and FIPV groups showed that CASP9 and BAX were significantly upregulated (*p* < 0.05), BCL2L1 and EGFR were significantly downregulated (*p* < 0.05), and AKT2 was downregulated but not significantly in the FIPV/control comparison group (Figure 5D). In the treatment/control comparison group, AKT2, EGFR, and PIK3CA were significantly upregulated (*p* < 0.05), and CASP9 was significantly downregulated (*p* < 0.05). In the treatment/FIPV comparison group, AKT2, PIK3CA, and EGFR were significantly upregulated (*p* < 0.05), BCL2L was upregulated but not significant, CASP9 was significantly downregulated (*p* < 0.05), and BAX was downregulated but not significant. The quantitative results of 4D-LFQ proteomics showed that compared with those in the FIPV group, EGFR, PIK3CA, and AKT2 were significantly upregulated in the treatment group (*p* < 0.05), and AKT3 and BCL2L1 tended to increase, CASP9 tended to significantly decrease (*p* < 0.05), and Bax tended to decrease (Figure 5E). The above results indicated that baicalin might inhibit FIPV infection by regulating the PI3K-AKT signaling pathway and the apoptosis pathway.

### 2.7. Identification of Key Proteins in the PI3K-AKT Signaling Pathway and Apoptosis Pathway by Western Blot

**Figure 6:**
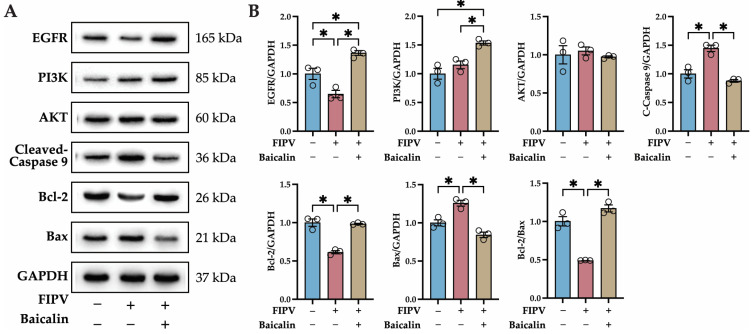
Identification of key proteins in the PI3K-AKT signaling pathway and apoptosis pathway by WB. (**A**) WB protein bands of EGFR, PI3K, AKT, C-caspase 9, Bcl-2, Bax, and GAPDH; (**B**) WB quantitative analysis of EGFR, PI3K, AKT, C-caspase 9, Bcl-2, and Bax relative to GAPDH; and results of Bcl-2/Bax relative expression. The data are represented as the mean ± SEM, *n* = 3, * *p* < 0.05.

According to the 4D-LFQ proteomics results, baicalin may inhibit FIPV infection by modulating the PI3K-AKT signaling pathway and the apoptosis pathway. To validate the results, the protein expression of the key proteins of the PI3K-AKT signaling pathway—EGFR, PI3K, and AKT—and the key proteins of the apoptosis pathway—caspase 9, Bcl-2, and Bax—were detected via Western blot (WB), and the relative expression levels were calculated using GAPDH as an internal reference protein. EGFR, PI3K, and Bcl-2 were significantly upregulated (*p* < 0.05); cleaved caspase 9 and Bax were significantly downregulated (*p* < 0.05); and Bcl-2/Bax was significantly upregulated (*p* < 0.05) in the treatment group compared with the FIPV group (Figure 6). This WB result was consistent with the trend of the quantitative 4D-LFQ proteomics results.

## 3. Discussion

Before the screening assay, we conducted a pretest for the toxicity of DMSO on CRFK cells. The MNTC of DMSO on CRFK cells was 0.83% (72 h incubation time), and the concentrations of DMSO for the compounds that were solubilized using DMSO and diluted with MM to MNTCs were all lower than 0.83%, so the interference of DMSO with the results of the assay could be excluded. In the present study, baicalin was screened from nine natural compounds with antiviral activity using a pharmacological viral inhibition assay, and baicalin was found to inhibit FIPV infection in vitro. Baicalin is a natural compound that has been shown to inhibit influenza virus infection via the caspase-3/GSDME pathway and to inhibit influenza A (H1N1) influenza-induced alveolar epithelial cell pyroptosis [17]. It can also inhibit the expression of toll-like receptor 4 through the PI3K/AKT/FoxO1 pathway, which plays a role in suppressing neuroinflammation [18]. FIPV belongs to the coronavirus genus, and research has shown that baicalin also has a positive inhibitory effect on coronaviruses by targeting and inhibiting coronavirus spike proteins to affect viral replication [19] and by targeting host mitochondrial OXPHOS to inhibit viral infection [20]. FIPV can induce apoptosis [21]; therefore, we examined the effect of baicalin on FIPV-induced apoptosis and found that treatment with baicalin resulted in a significant reduction in FIPV-induced apoptosis, a finding consistent with that obtained by Yu et al. [22] in a study of the baicalin-mediated inhibition of H9c2 cell apoptosis induced by hypoxia. In this study, apoptosis was detected by an Annexin V-FITC/PI assay, and early apoptosis (shown by green fluorescence) was detected; however, late apoptosis (shown by red fluorescence) was not detected, which may be related to the short infection time, so the subsequent infection time had to be optimized. Another thing that should be noticed is that baicalin, as a compound, may produce cytotoxicity and apoptosis. In this study, apoptosis, as reflected by green fluorescence, was significantly reduced after treatment of FIPV-infected CRFK cells with baicalin; however, whether the trace green fluorescence shown is apoptosis due to cytotoxicity produced by baicalin needs to be further investigated.

The prediction of drug action targets through the NP and MD has been well researched [23,24]. Proteomics is also a powerful tool for analyzing DEPs and understanding the underlying pharmacological mechanisms of TCM [25]. In this study, PubChem and GeneCards were used to screen the potential targets of baicalin for inhibiting FIPV infection. In screening the key targets, to avoid the computational bias of a single algorithm, nine computational methods in cytoHubba, which are based on the PPI network, were used for joint analysis [26] to accurately screen baicalin’s key targets for inhibiting FIPV infection. Molecular docking was then used to analyze the binding free energy and hydrogen bonding between the compounds and proteins, through which AKT1 was ultimately identified as the best target of baicalin for inhibiting FIPV infection. Then, we measured the cellular viral load to determine the optimal time for baicalin to exert its inhibitory effect before performing 4D-LFQ proteomics analysis and subjecting the samples to proteomics, which increased the accuracy of the 4D-LFQ proteomics analysis. Through 4D-LFQ proteomics analysis, it was found that the DEPs were strongly enriched according to GO functional enrichment, which made it inconvenient to screen the key DEPs and discuss their mechanism of action. Duan et al. [27] encountered a similar problem in the proteomics analysis of antiviral drugs; and for the subsequent mechanistic study, the researcher chose to analyze and validate the results of KEGG pathway enrichment. Therefore, to narrow the range of DEPs, the mechanism was also investigated from the KEGG pathway enrichment results in this study. The preliminary experimental results of this study demonstrated that baicalin inhibited FIPV-induced apoptosis and that AKT1 was the best target of baicalin for inhibiting FIPV infection. AKT1 is an isoform of the serine/threonine protein kinase AKT, with a protein size of 60 kDa; this protein is encoded by PKBα and is widely expressed in a wide range of tissues [28]. Research has shown that the PI3K-AKT signaling pathway, with AKT as the key protein, plays an important role in inhibiting viral infections [29,30]; therefore, combined with the results of KEGG enrichment analysis by 4D-LFQ proteomics, the PI3K-AKT signaling pathway and the apoptosis pathway were selected for subsequent studies.

The PI3K-AKT signaling pathway regulates a variety of target proteins to affect apoptosis. Studies have shown that activation of the PI3K-AKT signaling pathway inhibits the expression of p53, which, in turn, inhibits spontaneous apoptosis in B cells [31]. It can also reduce apoptosis by upregulating HIF-1α through the activation of PI3K-AKT and NF-κB [32]. Relevant research has shown that the PI3K-AKT signaling pathway can directly affect the expression of downstream apoptosis-related proteins. In a study on non-small cell lung cancer, PGRN was found to promote the expression of the antiapoptotic protein Bcl-2 by activating the PI3K-AKT signaling pathway [33]. Lin et al. [34] reported that it promoted the apoptosis of rectal cancer cells by inhibiting the activation of the PI3K-AKT signaling pathway, which, in turn, promoted the increase in cleaved caspase 9/caspase 9 and cleaved caspase 3/caspase 3. In recent years, baicalin has been shown to play a role in inhibiting apoptosis by modulating the PI3K-AKT pathway [35]. Zhang et al. [36] showed that baicalin could reverse the expression of tight junction proteins (occludin and ZO-1) and apoptosis-associated proteins (Bax, Bcl-2, caspase-3, and NF-κB) by activating the PI3K/Akt/mTOR signaling pathway, thereby inhibiting blood-spinal cord barrier permeability and reducing neuronal apoptosis after spinal cord injury. Zhao et al. [37] reported that baicalin could inhibit N-methyl-D-aspartate (NMDA)-induced apoptosis, autophagy, and oxidative stress in retinal ganglion cells (RGCs) by activating the PI3K-AKT signaling pathway in vitro and in vivo, thereby slowing pathological changes in the retinal tissues of glaucoma mice.

These studies demonstrated that baicalin can regulate the PI3K-AKT signaling pathway and that the PI3K-AKT signaling pathway can negatively regulate apoptosis. Based on these studies, and combining the heatmap and fold change results of the PI3K-AKT signaling pathway and the apoptosis pathway, we selected key proteins in two pathways—EGFR, PI3K, AKT, caspase 9, Bcl-2, and Bax—for validation. The results of volcano maps and quantitative 4D-LFQ proteomics analyses showed that there was a tendency for activation of the PI3K-AKT signaling pathway and a tendency for the inhibition of the apoptosis pathway in the baicalin-treated group compared to the FIPV group. Western blotting was then performed to validate the putative pathways involved, and the results showed that the PI3K-AKT signaling pathway was activated and that the apoptosis pathway was inhibited in FIPV-infected cells treated with baicalin. This result was consistent with the trend of the quantitative 4D-LFQ proteomics results, which demonstrated that baicalin activated the PI3K-AKT signaling pathway and inhibited the apoptosis pathway, similar to the results of previous studies [31,32,33,34,35,36,37]. However, as mentioned earlier, it has been shown in numerous studies that baicalin can inhibit apoptosis through cytotoxicity or through other signaling pathways; thus, the targeted and specific regulation of the PI3K-AKT signaling pathway and the caspase 9-induced apoptosis pathway by baicalin needs to be further investigated.

## 4. Materials and Methods

### 4.1. Virus, Cells, Compounds, and Antibodies

#### 4.1.1. Virus

The FIPV (BJ-01) was preserved by the Chinese Institute of Veterinary Drug Control (IVDC). The TCID_50_ of FIPV (BJ-01) was 10^−5.34^/0.1 mL. In this study, FIPV (BJ-01) was abbreviated as FIPV, and FIPV was added at a final concentration of 100 TCID_50_/0.1 mL.

#### 4.1.2. Cells

CRFK cells were preserved by IVDC. CRFK cells were cultured in a complete medium (DMEM supplemented with 10% fetal bovine serum (FBS) and 100 U/mL penicillin and streptomycin) in aerated cell culture flasks supplemented at 37 °C with 5% CO_2_. The maintenance medium (MM) used in the experiments was DMEM supplemented with 2% FBS and 100 U/mL penicillin and streptomycin. DMEM and 100 U/mL penicillin and streptomycin were purchased from Thermo Fisher Scientific Inc. (Waltham, MA, USA), and the FBS was purchased from Vazyme (Nanjing, China).

#### 4.1.3. Compounds

Baicalin, matrine, ligustrazine hydrochloride, caffeic acid, glycyrrhizic acid, puerarin, chlorogenic acid, tanshinone IIA, and dihydrotanshinone I were purchased from Shanghai Yuanye Bio-Technology Co., Ltd. (Shanghai, China), and their purity was 98% according to HPLC. GS-441524 was purchased from MCE (Monmouth Junction, NJ, USA), and its purity was 99.50% according to LC-MS. Baicalin, matrine, ligustrazine hydrochloride, and caffeic acid were dissolved in MM, and we used vortex vibration for solubilization; the final concentration was 2.0 mg/mL. Chlorogenic acid’s dissolution mode was the same as above, and the final concentration was 4.0 mg/mL. Glycyrrhizic acid and GS-441524 were dissolved in DMOS and then diluted with MM using vortex vibration for solubilization; the final concentration was 2.0 mg/mL and 1.2 mg/mL, respectively. Puerarin and dihydrotanshinone I’s dissolution modes were the same as above; then, ultrasonic methods were used for solubilization; the final concentrations were 1.0 mg/mL and 4.0 mg/mL, respectively. Tanshinone IIA was dissolved in MM, and we used vortex vibration and ultrasonic methods for solubilization; the final concentration was 4 mg/mL.

#### 4.1.4. Antibodies

All the antibodies were purchased from Proteintech (Wuhan, China) and diluted with TBST (Solarbio, Beijing, China). The dilution ratios of the EGFR monoclonal antibody, the PI3 kinase p85 alpha monoclonal antibody, the AKT polyclonal antibody, the caspase 9/p35/p10 monoclonal antibody, the Bcl2 monoclonal antibody, the BAX polyclonal antibody, the GAPDH monoclonal antibody, the HRP-conjugated Affinipure Goat Anti-Rabbit IgG (H+L), and the HRP-conjugated Affinipure Goat Anti-Mouse IgG (H+L) are 1:20,000, 1:3000, 1:6000, 1:5000, 1:2000, 1:20,000, 1:8000, 1:200,000, 1:8000, and 1:8000, respectively.

### 4.2. Screening of Natural Compounds That Inhibit FIPV Infection In Vitro

#### 4.2.1. Cytotoxicity Assay of the Compounds

CRFK cells were seeded in 96-well cell culture plates at a density of 1 × 10^4^ cells/well and incubated at 37 °C with 5% CO_2_ until the cell density reached more than 90%. Then, the medium was discarded, and the cells were washed twice with PBS. The compound to be tested was diluted in 10 successive gradients with MM at a 2-fold multiplicity (at the same time, there was a cell control group of 100 μL of MM, and 3 replicate wells were set for each concentration and group) and incubated at 37 °C with 5% CO_2_ for 72 h; then, the liquid in the plate was discarded and washed twice with PBS. Afterwards, the cytopathic ratios (CRs) of the compounds to cells were calculated according to the instructions of the Cell Counting Kit-8 (MCE, NJ, USA), and the CC_50_s of the compounds and the MNTCs at which the compounds could survive more than 90% of the cells were calculated using GraphPad Prism 10. In subsequent experiments, the final concentrations of the compounds after addition were the MNTCs of the compounds unless otherwise specified.

#### 4.2.2. Inhibitory Effects of Compounds on FIPV-Infected Cells

According to previous methods [38], CRFK cells were seeded in 96-well cell culture plates at a density of 1 × 10^4^ cells/well and cultured at 37 °C in 5% CO_2_ until the cell density was greater than 90%. After the cells were washed twice with PBS, 50 μL of test compound and 50 μL of FIPV were added to each well simultaneously (at the same time, a cell control group with only 100 μL of MM and a viral control group with only 100 μL of FIPV were added). There were 3 replicate wells for each compound and group. After 72 h of incubation at 37 °C and 5% CO_2_, the liquid in the plate was discarded, and the cells were washed twice with PBS. The MIR of the compounds to the virus was calculated according to the instructions of the Cell Counting Kit-8. Afterwards, the compounds with MIR > 50% were diluted to 8 concentrations with MM, and the above process was repeated. Then, the EC_50_ values of the compounds were calculated with GraphPad Prism 10. Finally, among the compounds satisfying both IR > 50% and CC_50_/EC_50_ (selection index, SI) > 3, the compound with the largest IR was selected for subsequent experiments.

### 4.3. Impact of the Compound on FIPV Replication, Adsorption, and Direct Inactivation

#### 4.3.1. Impact of the Compound on FIPV Replication

According to previous methods [39], CRFK cells were seeded in 96-well cell culture plates at a density of 1 × 10^4^ cells/well and cultured at 37 °C in 5% CO_2_ until the cell density was greater than 90%. Then, the medium was discarded, and the cells were washed twice with PBS. FIPV was first added into 96-well cell culture plates at 100 μL/well then incubated at 37 °C and 5% CO_2_ for 1, 2, 4, 6, 8, 10, 12, and 14 h, respectively. After that, the viral solution was discarded and washed with PBS twice and then the compound was added at 100 μL/well and incubated at 37 °C and 5% CO_2_ (at the same time, a cell control group with only 100 μL of MM and a viral control group with only 100 μL of FIPV were added). There were 3 replicate wells for each compound and group. After the CPE of the viral control group reached more than 80%, the liquid in the culture plate was removed and washed twice with PBS. Afterwards, the IR of the compounds against the virus was computed according to the directions of the Cell Counting Kit-8.

#### 4.3.2. Impact of the Compound on FIPV Adsorption

According to previous methods [40], CRFK cells were seeded in 96-well cell culture plates at a density of 1 × 10^4^ cells/well and cultured at 37 °C in 5% CO_2_ until the cell density was greater than 90%. Then, the medium was discarded, and the cells were washed twice with PBS. The compound was first added into 96-well cell culture plates at 100 μL/well and then incubated at 37 °C and 5% CO_2_ for 0.25, 0.2, 1, 2, 4, and 6 h, respectively, then incubated at 4 °C for 1 h. After that, the liquid in the plates was removed and washed with PBS twice. Then, added FIPV into cells at 100 μL/well and incubated at 37 °C and 5% CO_2_ (at the same time, a cell control group with only 100 μL of MM and a viral control group with only 100 μL of FIPV were added). There were 3 replicate wells for each compound and group. After the CPE of the viral control group reached more than 80%, the liquid in the culture plate was removed and washed twice with PBS. Afterwards, the IR of the compounds against the virus was computed according to the directions of the Cell Counting Kit-8.

#### 4.3.3. Impact of the Compound on FIPV Direct Inactivation

According to previous methods [41], CRFK cells were seeded in 96-well cell culture plates at a density of 1 × 10^4^ cells/well and cultured at 37 °C in 5% CO_2_ until the cell density was greater than 90%. Then, the medium was discarded, and the cells were washed twice with PBS. The compound was mixed with FIPV in the ratio of 1:1 and incubated at 37 °C and 5% CO_2_ for 30, 60, 90, 120, and 150 min, respectively; then, the mixture of different incubation durations were added into 96-well cell culture plates at 100 μL/well and incubated at 37 °C and 5% CO_2_, respectively (at the same time, a cell control group with only 100 μL of MM and a viral control group with only 100 μL of FIPV were added). There were 3 replicate wells for each compound and group. After the CPE of the viral control group reached more than 80%, the liquid in the culture plate was removed and washed twice with PBS. Afterwards, the IR of the compounds against the virus was computed according to the directions of the Cell Counting Kit-8.

### 4.4. Effect of the Compounds on FIPV-Induced Apoptosis

CRFK cells were seeded in 96-well cell culture plates at a density of 1 × 10^4^ cells/well and cultured at 37 °C in 5% CO_2_ until the cell density was greater than 90%. After the cells were washed twice with PBS, 50 μL of test compound and 50 μL of FIPV were added to each well simultaneously (at the same time, a cell control group with only 100 μL of MM and a viral control group with only 100 μL of FIPV were added). Three replicate wells were used for each group. After 72 h of incubation at 37 °C and 5% CO_2_, the liquid in the plate was discarded, and the cells were washed twice with PBS. Then, apoptosis was detected according to the instructions of the Annexin V-FITC/PI Apoptosis Detection Kit (MCE, USA). The results were observed using a fluorescence inverted microscope, and the fluorescence intensity was analyzed using ImageJ.

### 4.5. NP and MD Were Used to Determine the Best Targets of the Compounds for Inhibiting FIPV Infection

The 3D structure of the compound was obtained through PubChem (https://pubchem.ncbi.nlm.nih.gov/, accessed on 20 November 2023) [42] and later uploaded to PharmMapper (http://www.lilab-ecust.cn/pharmmapper/, accessed on 21 November 2023) [43,44,45] to obtain the potential targets on which the compound acts. The pathogenic targets possibly related to FIPV were obtained from GeneCards (https://www.genecards.org/, accessed on 21 November 2023) [46,47] using “Coronavirus” as a keyword [48,49] (selected the top 300 sorted by GeneCards Inferred Functionality Scores). The above-collected targets were unified and named by UniProt (https://www.uniprot.org/, accessed on 21 November 2023) [50] and then intersected to obtain the potential targets of the compounds that inhibit FIPV infection. These potential targets were uploaded to Metascape (https://metascape.org/, accessed on 22 November 2023) [51] for GO function and KEGG pathway enrichment analysis. Moreover, these potential targets were uploaded to STRING (https://cn.string-db.org/, accessed on 22 November 2023) [52] to obtain the node information of the PPI network. The node information was imported into Cytoscape 3.9.0 (National Resource for Network Biology, USA) [53] and analyzed by CytoNCA 2.1.6 [54] to construct a PPI network. The topological analysis was also performed using 9 analysis methods (betweenness, closeness, degree, EPC, MCC, MNC, radiality, bottleNeck, and stress) in cytoHubba [55], and the key targets were obtained by filtering the targets ranked in the top 5 of each analysis method. Afterwards, the 3D structure files of the key targets were obtained by UniProt and imported into UCSF Chimera 1.16 (RBVI, San Francisco, CA, USA), and molecular docking with the compounds was carried out using AutoDock Vina 1.1.2, after which the conformations with the lowest binding free energies were selected for the hydrogen bonding analyses, and the best targets were identified by comparing the binding free energies and the number of hydrogen bonds of each compound-target.

### 4.6. Determination of the Cellular Viral Load at Different Times of Compound Action

CRFK cells were seeded in a 96-well cell culture plate at a density of 1 × 10^4^ cells/well and cultured at 37 °C in 5% CO_2_ until the cell density was greater than 90%. After the cells were washed twice with PBS, 50 μL of compound and 50 μL of FIPV were added to each well simultaneously, and the plates were incubated at 37 °C and 5% CO_2_ for 12, 24, 36, 48, 60, and 72 h (at the same time, a cell control group with only 100 μL of MM and a viral control group with only 100 μL of FIPV were added). There were 3 replicate wells for each group and incubation time. After the liquid in the plate was discarded, the cells were washed twice with PBS. Then, the cells were digested by adding tryptic digest at 30 μL/well, the cell suspension was collected, and the cell precipitates were collected by centrifugation at 3000 rpm for 1 min. Total cellular RNA was extracted after the cell precipitates were resuspended in PBS according to the instructions of the FastPure Viral DNA/RNA Mini Kit Pro (Vazyme, Nanjing, China). Then, reverse transcription was performed using HiScript IV RT SuperMix for qPCR (+gDNA wiper) (Vazyme, Nanjing, China). The sequences of the primers used for the detection of the FIPV-N (GenBank: MK840958.1) were TGCTTCGGCTAACTTTGGTG (F: 5′→3′) and CAATCATCTCAACCTGTGTGTCAT (R: 5′→3′). The sequences of the primers used for the detection of the GAPDH genes were AGGTCGGTGTGAACGGATTT (F: 5′→3′) and TGCCGTGGGTGGAATCATAC (R: 5′→3′). The qPCR system was established using Taq Pro Universal SYBR qPCR Master Mix (Vazyme, Nanjing, China), and the reaction was carried out using Roche LightCycler 480 (Roche, Basel, Switzerland) according to the manufacturer’s instructions. Finally, GAPDH was used as the reference gene, and the obtained CT value was used to calculate the relative expression of the FIPV-N gene via the 2^−ΔΔCT^ method to compare the viral content of FIPV at different infection times.

### 4.7. 4D-LFQ Proteomics Analysis of the Mechanisms by Which Compounds Inhibit FIPV Infection

According to the methods of Section 4.6, post-infection cells were collected at the appropriate time of infection and then quickly frozen in liquid nitrogen. The cell control group was named the control group (Group C), the virus control group was named the FIPV group (Group F), the compound treatment group was named the treatment group (Group T), and each group contained three replicate samples. Afterwards, they were sent to Shanghai Applied Protein Technology Co., Ltd. (Shanghai, China) for 4D-LFQ proteomics analysis. Visual mapping was performed based on the analysis results, and the mechanism by which the compound inhibited FIPV infection was determined by combining the results of existing experiments.

### 4.8. Verification of Key Proteins of the Pathway Using Western Blot

Based on the results of the above experiments, the cell precipitates were collected according to the method in Section 4.6 and grouped as described in Section 4.7. After infection, the cells were collected at the appropriate time of infection, and the proteins were extracted from the samples according to the instructions of a total protein extraction kit (strong) (Solarbio, Beijing, China). Afterwards, the protein concentration of the extracted samples was determined separately using a BCA Protein Quantification Kit (Vazyme, Nanjing, China), and all sample proteins were diluted to the same concentration using PBS. The diluted protein samples were mixed with NuPAGE LDS Sample Buffer (4×) (Invitrogen, Waltham, MA, USA) at a ratio of 1:4 and heated at 70 °C for 10 min to denature the proteins. After that, the denatured proteins were added to 10 wells of 12% sodium dodecyl sulfate-polyacrylamide gel electrophoresis (SDS-PAGE) (Vazyme, Nanjing, China) at 20 μg/well, after which the total proteins were separated on NuPAGE MES SDS Running Buffer (Invitrogen, CA, USA). After separation, proteins were transferred from the gel onto 0.45 μm polyvinylidene fluoride (PVDF) membranes (Millipore, Billerica, MA, USA) using WB Transfer Buffer (Solarbio, Beijing, China). After that, the PVDF membranes were blocked with 5% skim milk (Solarbio, Beijing, China) at room temperature for 2 h. At the end of the closure, the membranes were washed with TBST (6 times, each time for 5 min, as described below); then. the primary antibodies were added and incubated at 4 °C overnight. The next day, the PVDF membranes were washed with TBST, the primary antibodies were removed, and the secondary antibodies were added and incubated at room temperature for 2 h (the antibody information and dilutions are shown in Section 4.1.4). At the end of the incubation, the PVDF membranes were washed with TBST, after which the protein bands were exposed and imaged using a SuperPico ECL Chemiluminescence Kit (Vazyme, Nanjing, China) and an Al680 imaging system (GE, Boston, MA, USA). The gray values of the WB bands were analyzed using ImageJ 1.54f, and the relative expression was calculated using GAPDH as an internal reference protein.

### 4.9. Statistical Analyses

The NP and 4D-LFQ proteomics results were visualized and mapped using the Hiplot (https://hiplot.com.cn/home/index.html, accessed on 10 December 2023) and bioinformatics (https://www.bioinformatics.com.cn/, accessed on 10 December 2023) online platforms. All remaining data were analyzed using GraphPad Prism 10 (GraphPad Software, Inc., Boston, MA, USA) software, and the results are presented as the mean ± SEM. One-way ANOVA was used to compare the differences between groups. Different lowercase letters and “*” indicate significant differences (*p* < 0.05).

## 5. Conclusions

Baicalin has an anti-FIPV effect in vitro, and it can inhibit apoptosis by regulating the PI3K-AKT signaling pathway and the apoptosis pathway, which, in turn, inhibits FIPV infection.

## Figures and Tables

**Table 1:** Cytotoxicity test results.

S/N	Compounds	MNTC (μg/mL)	CC_50_ (μg/mL)
1	Baicalin	31.36	114.5
2	Matrine	699.8	1110
3	Ligustrazine hydrochloride	969.6	2709
4	Caffeic acid	53.43	58.78
5	Glycyrrhizic acid	1412	3324
6	Puerarin	83.77	208.8
7	Chlorogenic acid	1605	2170
8	Tanshinone IIA	290.9	806.6
9	Dihydrotanshinone I	246.4	426.4
10	GS-441524	36.20	70.31

**Table 2:** Potential targets of baicalin for the inhibition of FIPV infection.

S/N	UniProt ID	Gene Name	Protein Name
1	P31749	AKT1	RAC-alpha serine/threonine-protein kinase
2	P08758	ANXA5	Annexin A5
3	P13501	CCL5	C-C motif chemokine 5
4	P23946	CMA1	Chymase
5	P35221	CTNNA1	Catenin alpha-1
6	P43235	CTSK	Cathepsin K
7	P27487	DPP4	Dipeptidyl peptidase 4
8	P00533	EGFR	Epidermal growth factor receptor
9	P03372	ESR1	Estrogen receptor
10	P60568	IL2	Interleukin-2
11	P02788	LTF	Lactotransferrin
12	P14555	PLA2G2A	Phospholipase A2, membrane associated
13	P62937	PPIA	Peptidyl-prolyl cis-trans isomerase A
14	P18031	PTPN1	Tyrosine-protein phosphatase non-receptor type 1
15	P02753	RBP4	Retinol-binding protein 4
16	P12724	RNASE3	Eosinophil cationic protein
17	P16109	SELP	P-selectin

## Data Availability

The data generated/analyzed during the current study are available from the corresponding author upon request.

## Abbreviations

The following abbreviations are used in this manuscript:

FIP	Feline infectious peritonitis
FIPV	Feline infectious peritonitis virus
CRFK	Crandell Reese feline kidney (cell)
MNTC	Maximum noncytotoxic concentration
CC50	50% cytotoxic concentration
EC50	50% effective concentration
SI	Selection index
MIR	Maximum inhibition ratio
NP	Network pharmacology
MD	Molecular docking
4D-LFQ	4D label-free quantitative
TCM	Traditional Chinese medicine
PPI	Protein-protein interaction
DEPs	Differentially expressed proteins
BP	Biological process
CC	Cellular component
MF	Molecular function
WB	Western blot
FBS	Fetal bovine serum
MM	Maintenance medium
